# Supplementary figures and images for: Effects of Different Amounts of Corn Silk Polysaccharide on the Structure and Function of Peanut Protein Isolate Glycosylation Products
Source: Foods. 2022 Jul 26;11(15):2214. doi: 10.3390/foods11152214 (PMC9330836; doi:10.3390/foods11152214)

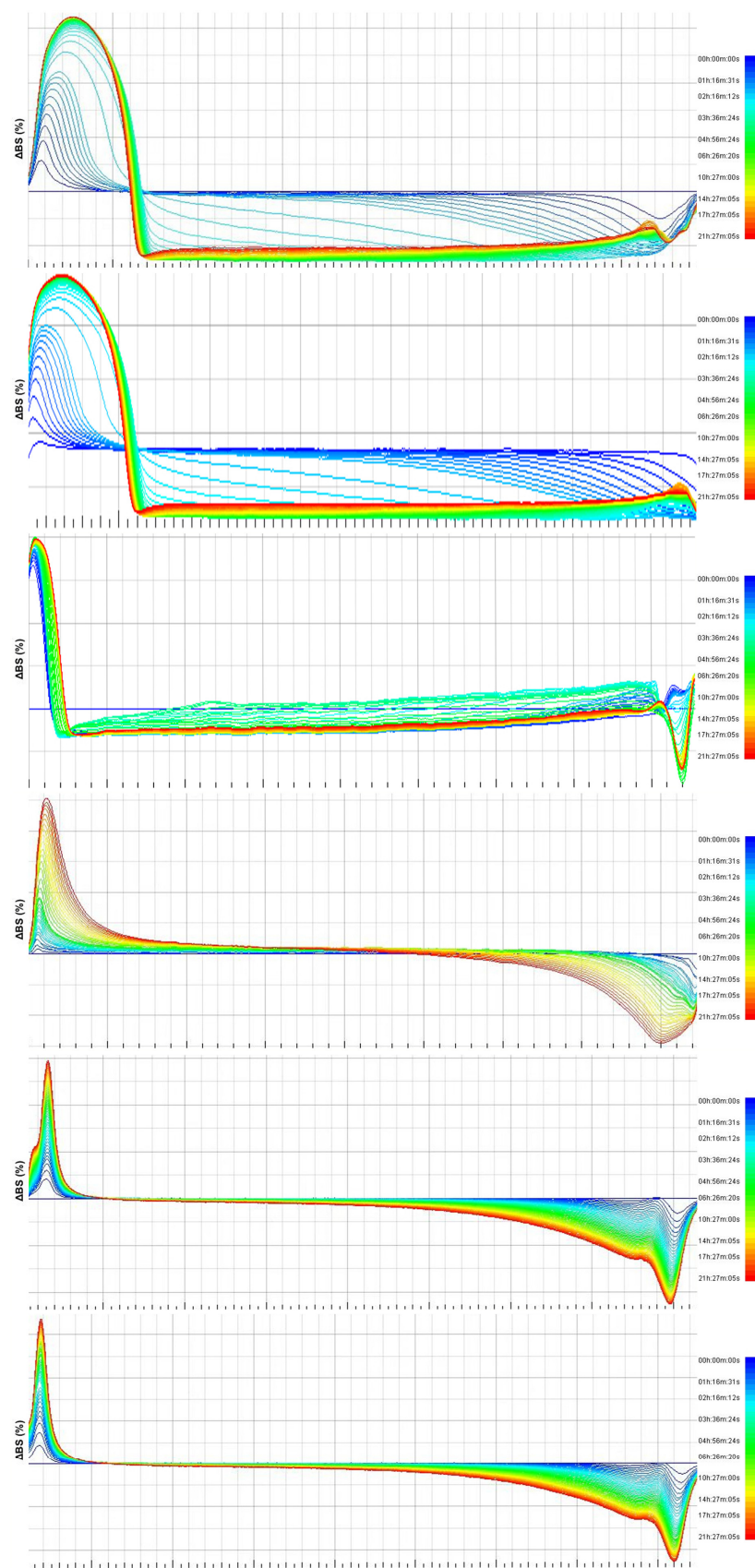

**Figure S1.** Delta backscattering profiles of PPI-CSP complexes aqueous solutions.

Supplement: Supplementary file 1 [file foods-11-02214-s001.zip › foods-1811177-supplementary.pdf]
